# High-Performance Flexible Asymmetric Supercapacitor Based on CoAl-LDH and rGO Electrodes

**DOI:** 10.1007/s40820-017-0134-8

**Published:** 2017-02-18

**Authors:** Shuoshuo Li, Pengpeng Cheng, Jiaxian Luo, Dan Zhou, Weiming Xu, Jingwei Li, Ruchun Li, Dingsheng Yuan

**Affiliations:** 1 School of Chemistry and Materials Science, Jinan University, Guangzhou, 510632 People’s Republic of China; 2 Guangzhou Key Laboratory of Vacuum Coating Technologies and New Energy Materials, Jinan University, Guangzhou, 510632 People’s Republic of China

**Keywords:** Flexible asymmetric supercapacitor, Layer double hydroxides, Reduced graphene oxide, Cycle stability

## Abstract

A flexible asymmetric supercapacitor (ASC) based on a CoAl-layered double hydroxide (CoAl-LDH) electrode and a reduced graphene oxide (rGO) electrode was successfully fabricated. The CoAl-LDH electrode as a positive electrode was synthesized by directly growing CoAl-LDH nanosheet arrays on a carbon cloth (CC) through a facile hydrothermal method, and it delivered a specific capacitance of 616.9 F g^−1^ at a current density of 1 A g^−1^. The rGO electrode as a negative electrode was synthesized by coating rGO on the CC via a simple dip-coating method and revealed a specific capacitance of 110.0 F g^−1^ at a current density of 2 A g^−1^. Ultimately, the advanced ASC offered a broad voltage window (1.7 V) and exhibited a high superficial capacitance of 1.77 F cm^−2^ at 2 mA cm^−2^ and a high energy density of 0.71 mWh cm^−2^ at a power density of 17.05 mW cm^−2^, along with an excellent cycle stability (92.9% capacitance retention over 8000 charge–discharge cycles).

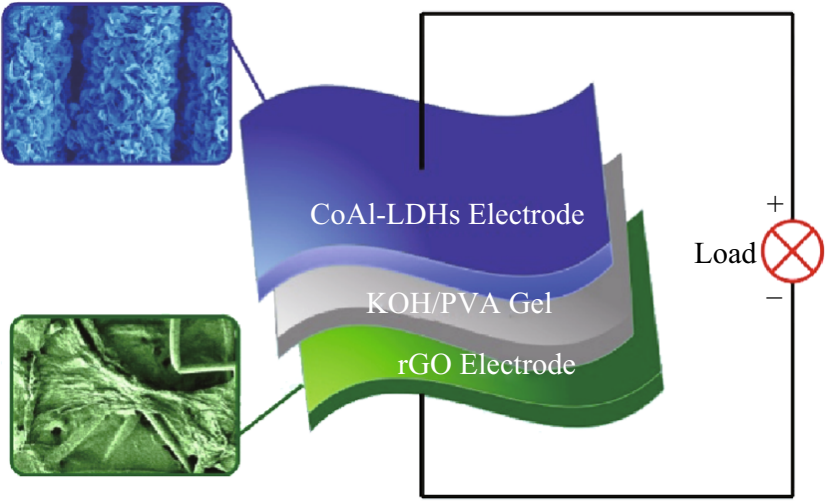

## Highlights

- CoAl-LDH (layer double hydroxide) electrode and binder-free rGO (reduced graphene oxide) electrode were successfully synthesized and assembled to produce a flexible ASC (asymmetric supercapacitor).
- The assembled ASC device exhibited excellent capacitive performance.

## Introduction

The increasing demand for portable electronic devices and hybrid vehicles has stimulated the development of high-performance flexible energy storage devices [1–5]. Flexible supercapacitors (SCs), which have played a key role in medical treatment, military, entertainment, and industry, are considered as potential candidates for flexible and portable energy storage devices because of their high flexibility and light weight [6–9]. Although SCs have desired properties such as fast charge–discharge rate, high power density, and cycling stability, the energy density of flexible SCs needs to be improved to meet the fast-growing market for portable electronic devices and the development of hybrid vehicles [10–15].

According to the equation of energy density (*E* = 0.5 CV^2^), the energy density can be enhanced by increasing the specific capacitance (*C*) or the voltage window (*V*) [16–19]. On the one hand, developing appropriate electrode materials favors enhancing the specific capacitance and correspondingly improving the energy density [20–22]. Based on the energy storage mechanisms, electrode materials can be classified into two types: electric double-layer capacitors (EDLC) and pseudocapacitors. The EDLC electrode material stores energy based on electrostatic charge accumulation on the surface between the electrode and electrolyte [23–26]. Carbon materials have mostly been used because of their high specific surface area, good electrical conductivity, and excellent stability. Among them, graphene, a single-atom-thick sheet of hexagonally arrayed *sp* ^2^-bonded carbon atoms, which has a high theoretical specific capacitance of 550 F g^−1^, has emerged as a promising candidate for electrode materials [27–30]. Pseudocapacitive electrode materials mainly rely on fast and reversible faradic reactions to store energy. Transition metal oxides and hydroxides have extensively been developed as electrode materials of this type. However, considering their poor electronic conductivity, various transition metal hydroxides and oxides combined with electrically conductive frameworks, such as a carbon cloth (CC) and Ni foam, have attracted increasing attention [31–33]. For example, Yu et al. have reported that NiFe_2_O_4_ nanoparticles can be directly grown on a flexible CC substrate by a facile surfactant-assisted hydrothermal method that showed good electrochemical properties [34]. This designed device possessed several advantages including flexibility, a binder-free process, and portability, which are more desirable for portable electronic devices.

On the other hand, constructing an asymmetric system with different positive and negative electrode materials can increase the voltage window and thus enhance the energy density. Fang et al. successfully fabricated a flexible coaxial-type fiber solid-state asymmetrical SC (ASC) based on Ni_3_S_2_ nanorod array electrodes and pen-ink electrode. Compared to symmetric SCs (SSCs) based on Ni_3_S_2_ electrodes, the ASC device provides an increased energy density [35]. Based on the above consideration, here, a flexible ASC device was designed, including CoAl-layered double hydroxide (CoAl-LDH) and reduced graphene oxide (rGO) grown on the CC as the positive electrode and the negative electrode, respectively. Accordingly, the CoAl-LDH electrode with ultrathin nanosheets and porous nanostructure showed a high specific capacitance of 616.9 F g^−1^ at a current density of 1 A g^−1^. As the negative electrode, rGO showed a specific capacitance of 110 F g^−1^ at a current density of 2 A g^−1^. When assembled together, the flexible ASC delivered a high capacitance of 1.77 F cm^−2^ at 2 mA cm^−2^ and a high energy density of 0.71 mWh cm^−2^ at a power density of 17.05 mW cm^−2^.

## Experimental Section

### Preparation of CoAl-LDH Nanosheet on CC (CC@CoAl-LDHs)

The CC (1.0 × 2.0 cm^2^) was cleaned with concentrated hydrochloric acid, acetone, and deionized water and then dried. The cleaned CC was immersed in 2.0 mol L^−1^ Co(NO_3_)_2_ ***·***6H_2_O solution for 1 min and then removed. The resulting CC was dried at 60 °C for 15 min. The whole procedure was repeated three times. The preprocessed CC was placed into a Teflon-lined autoclave with 2.0 mmol Co(NO_3_)_2_ **·**6H_2_O, 2.0 mmol Al(NO_3_)_3_, 4.0 mmol CO(NH_2_)_2_, 8.0 mmol NH_4_F, and 60.0 mL H_2_O. Then, the autoclave was sealed and heated in an oven at 140 °C for 16 h. After cooling down to 25 °C, the CC was washed with deionized water and alcohol several times and dried at 60 °C for 24 h.

### Preparation of rGO on CC (CC@ rGO)

GO was prepared using Hummer’s method. Aqueous GO solution (50.0 mL, 1.0 mg mL^−1^) was reacted in a Teflon-lined autoclave at 180 °C for 6 h. After cooling down to room temperature, the products were filtered, washed, and freeze-dried. Then, 10.0 mg rGO powders were dispersed in 10.0 mL N-methyl-2-pyrrolidone (NMP) and ultrasonicated for 1 h to form stable aqueous rGO. The CC was immersed in aqueous rGO for 2 min, removed, and dried at 60 °C for 5 min. The process was repeated 10 times to obtain CC@rGO.

### Materials Characterization

The samples were characterized using a MSAL-XD2 X-ray diffractometer (XRD, Cu Ka, 40 kV, 20 mA, λ = 1.5406 Å). The morphologies were examined by scanning electron microscopy (SEM, ZEISS Ultra 55) and field-emission transmission electron microscopy (FETEM, JEM2010) operating at 200 kV. Nitrogen sorption isotherms of the as-prepared materials were studied using a Micromeritics TriStar 3000 analyzer at 77 K. The plot of specific surface area was deduced from the isotherm analysis of the adsorption data at a relative pressure (P/P_0_) of 0–1.0, and the average pore diameters were collected from the peak value of the pore diameter distribution.

### Electrochemical Measurements

All electrochemical measurements were taken on an electrochemical workstation (CHI 660D, CH Instruments, Inc.) at room temperature in a conventional three-electrode system. A Ni foil and a Hg/HgO electrode were used as the counter and reference electrodes, respectively. The working electrode was measured by cyclic voltammetry (CV) and galvanostatic charge–discharge in a 6-M KOH aqueous solution.

### Flexible Asymmetric Supercapacitor Devices

The flexible asymmetric SC consists of CC@CoAl-LDHs as the positive electrode, CC@rGO as the negative electrode, and PVA/KOH as the electrolyte and separator. The assembly process is as follows: PVA/KOH gel was prepared by mixing 6 g of PVA powder into 100 mL of 6 M KOH aqueous solution. The mixture was heated to 90 °C with stirring until it became clear. Then, the positive and negative electrodes were immersed into the PVA/KOH gel for 15 min and then solidified for 10 min. Next, the electrodes were immersed in the gelled electrolyte again and assembled into a sandwich structure. The fabrication of a flexible ASC cell was completed after the gel electrolyte solidified at room temperature.

## Results and Discussion

### Positive Electrode

**Fig. 1:**
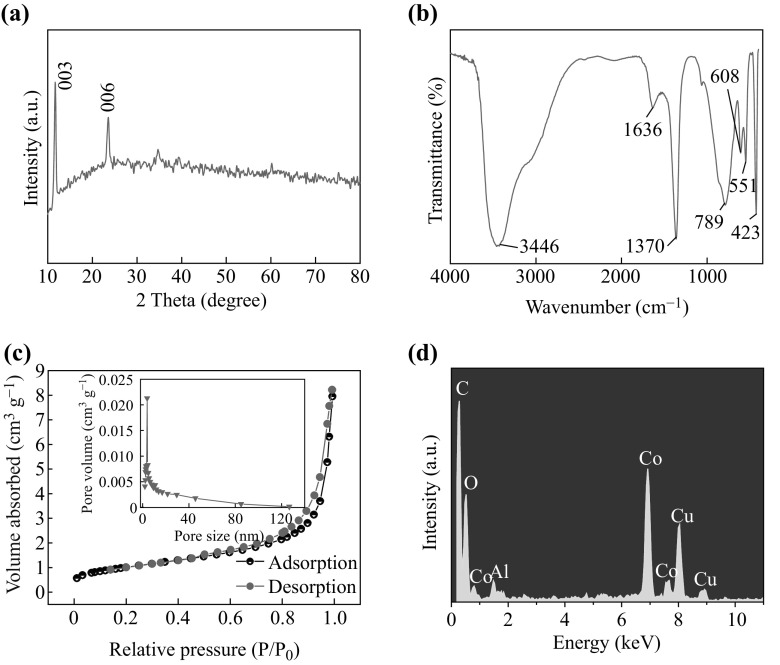
**a** XRD pattern; **b** FTIR; **c** N_2_ adsorption–desorption isotherms and pore sizes distribution (*inset*); **d** EDX spectroscopy of CoAl-LDHs

Figure 1a shows the XRD pattern of CoAl-LDHs. A strong peak appeared at 2*θ* = 12.2° and 23.9°, corresponding to the diffraction lattice of the (003) and (006) planes, respectively, which suggested that the as-prepared material had a hydrotalcite-type structure [17]. Fourier transform infrared spectroscopy (FTIR) of the as-prepared CoAl-LDHs is shown in Fig. 1b. The strong absorption peak at 3446 cm^−1^ corresponded to the O–H stretching vibration of the hydroxyl groups. Peaks at 1370 and 789 cm^−1^ corresponded to the *v*3 vibration and bending modes of CO_3_^2−^, respectively. In addition, the weak absorption at 1636 cm^−1^ was attributed to the bending vibration of water molecules. Other peaks below 800 cm^−1^ were related to the metal–oxygen (M–O) stretching and bending vibrations [36].

N_2_ adsorption and desorption isotherms were performed to analyze the porous structure and porous size distribution of the CoAl-LDH nanosheets, as shown in Fig. 1c. The sample presented a type III curve with a H1 hysteresis loop at a high relative pressure, demonstrating the presence of macropores and mesopores. The specific surface area of CoAl-LDHs can be calculated using the Brunauer–Emmett–Teller (BET) equation and could reach up to 45 m^2^ g^−1^. The Barrett–Joyner–Halenda (BJH) pore-size-distribution curve is shown in Fig. 1c (insets). A noticeable narrower peak of the pore size distribution could be observed and showed the desired pore size distribution at 4 nm. The abundant pores could potentially enhance electrolyte diffusion and improve the power capability of the obtained sample. Furthermore, the energy-dispersive X-ray spectroscopy (EDX) spectrum (Fig. 1d) revealed that the CoAl-LDH nanosheets mainly contained Co, Al, Cu, O, and C, while most of the C and Cu signals were from the carbon-supported and Cu grid. All these results indicate that CoAl-LDHs were successfully prepared.

**Fig. 2:**
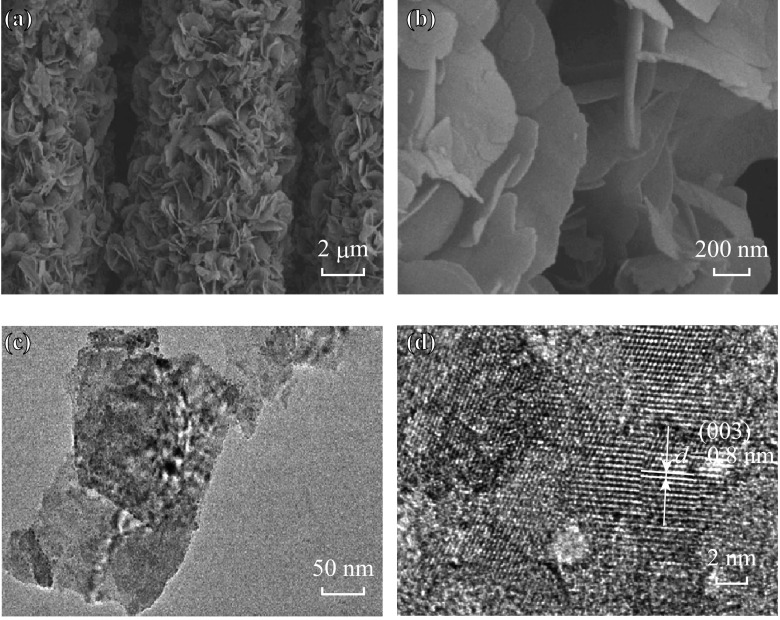
**a**, **b** SEM images of CoAl-LDHs on CC electrode and **c**, **d** TEM images of CoAl-LDHs

The morphologies of CC@CoAl-LDHs were characterized by SEM and TEM. Figure 2a shows the SEM image of CC@CoAl-LDHs. It is clearly observed that CoAl-LDHs uniformly covered the CC with a dense packing. The magnified SEM image in Fig. 2b revealed that the CoAl-LDHs are composed of around 10-nm-thick nanosheets, which could increase the specific surface area of CoAl-LDHs and yield excellent electrochemical properties. In addition, the TEM images of CoAl-LDHs scraped from the CC are displayed in Fig. 2c, d. Figure 2c shows the CoAl-LDH nanosheets corresponding to the SEM. The HRTEM image of the CoAl-LDH nanosheet (Fig. 2d) showed a lattice spacing of 0.8 nm, corresponding to the (003) plane.

**Fig. 3:**
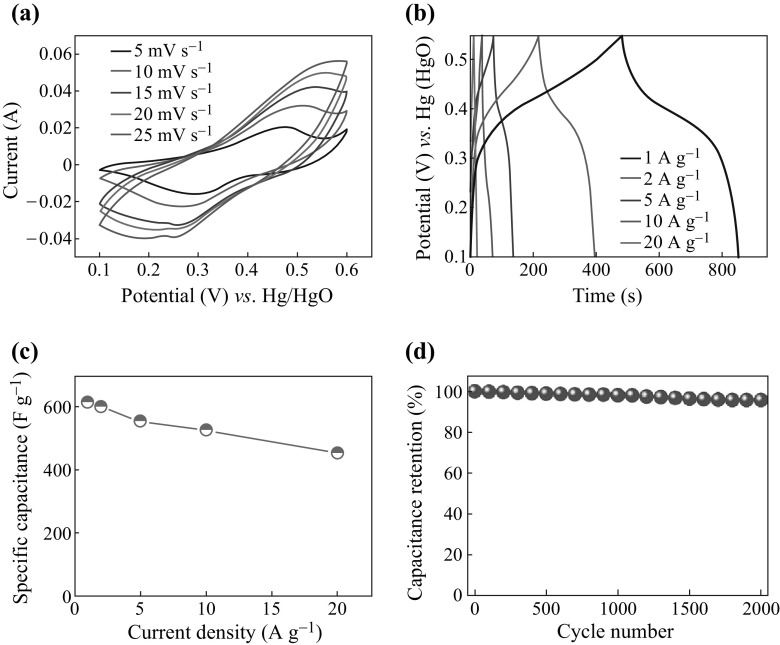
Electrochemical performances of the CoAl-LDHs@CC electrode: **a** CVs at different scan rates; **b** GCD curves at different current densities; **c** Plot of C sp versus current density; **d** Cycling performances during 2000 cycles at a large current density of 10 A g^−1^

To investigate the electrochemical performance of the CC@CoAl-LDHs, the CV curves were measured at different scan rates in 6 M KOH solution, as shown in Fig. 3a. A pair of redox peaks was present in every CV curve, which indicated that the CoAl-LDHs were typical pseudocapacitor materials. All the redox peaks were symmetrical at different scan rates, implying the excellent reversible redox reaction at/near the surface of the CoAl-LDH electrode. The galvanostatic charge–discharge curve of CoAl-LDHs at different current densities is shown in Fig. 3b. The presence of an obvious charge–discharge platform further evidenced that CoAl-LDHs possessed pseudocapacitance characteristics. Meanwhile, the specific capacitance of CoAl-LDHs in Fig. 3c could be calculated from the discharge time using the following equation:

1 $$C = \frac{it}{m\Delta V}$$

where *i*, *t*, *m*, and Δ*V* represent the discharge current (*A*), discharge time (*s*), mass of active materials (*g*), and total potential deviation (*V*), respectively. The CoAl-LDHs delivered a high specific capacitance of 616.9 F g^−1^ at a current density of 1 A g^−1^. Simultaneously, a capacitance of 454.4 F g^−1^ was still retained at a very high current density of 20 A g^−1^, indicating a good rate capability. These excellent electrochemical properties could be attributed to three factors: (1) The CoAl-LDHs uniformly covered on the surface of the CC could improve the electron transport from the active materials to the current collector; (2) the two-dimensional porous structure could accelerate the permeation of electrolyte for fast and reversible faradic reactions, increase the specific surface area of CoAl-LDHs and accordingly enhance the electrochemical properties; (3) the redox reaction between Co^2+^ and Co^3+^ in CoAl-LDHs could contribute more efficiently to the pseudocapacitance [37]. In addition, the long-term cycling stability of the CoAl-LDH electrode was evaluated by galvanostatic charge–discharge measurement for 2000 cycles at a current density of 10 A g^−1^. As shown in Fig. 3d, the specific capacitance of the CoAl-LDH electrode still remained 95.8% after 2000 cycles and suggested an excellent stability.

### Negative Electrode

**Fig. 4:**
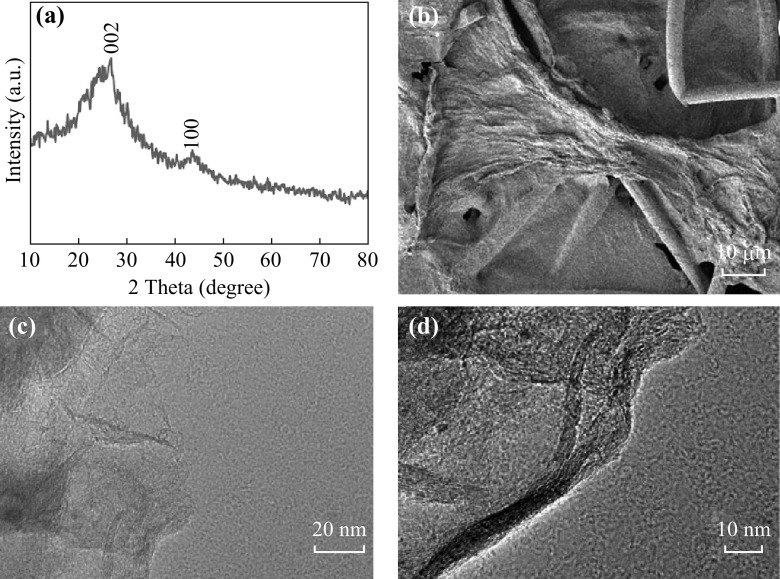
**a** XRD pattern of rGO@CC; **b** the SEM image of rGO@CC electrode; and (**c**, **d**) the TEM image of rGO

The XRD pattern of rGO is shown in Fig. 4a. It can clearly be observed that two diffraction peaks were located at 26° and 43°, associated with the (002) and (100) planes of carbon, respectively. Moreover, the morphologies and microstructures of rGO were investigated by SEM and TEM. Figure 4b shows that rGO was coated well on the surface of the CC substrate to form 3D architectures, which could be beneficial in reducing aggregation of the rGO. The TEM in Fig. 4c, d revealed that rGO possessed a rippled silk veil structure with a wrinkled shape.

**Fig. 5:**
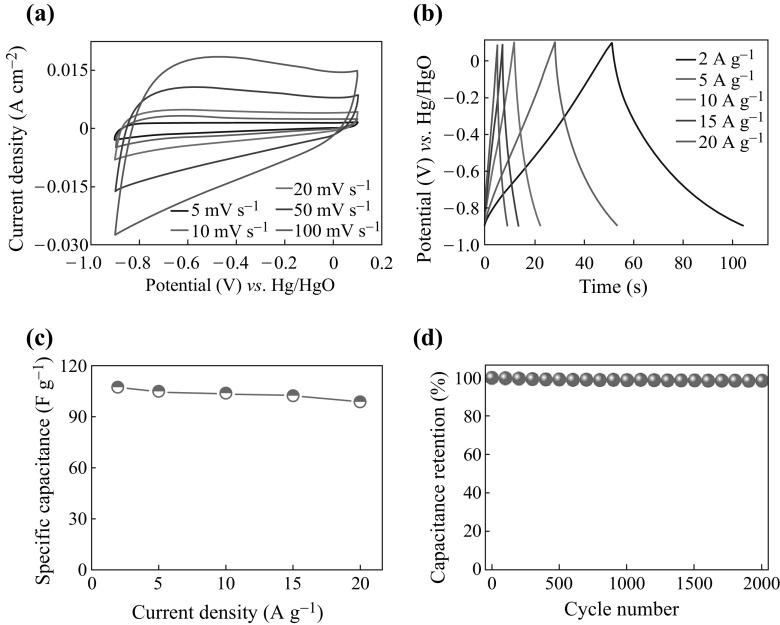
Electrochemical performances of the rGO@CC electrode: **a** CVs at different scan rates; **b** GCD curves at different current densities; **c** Plot of specific capacitance versus current density; **d** Cycling performance during 2000 cycles at a large current density of 10 A g^−1^

The electrochemical performance of rGO by CV and galvanostatic charge–discharge was measured in 6 M KOH aqueous solution over the potential range of 0.1 to −0.9 V. Figure 5a shows the CV curves of rGO at various scan rates from 5 to 100 mV s^−1^. The quasi-rectangular shape indicated its excellent electric double-layer capacitance. In particular, the shape of the CV curves did not change under a fast or slow scanning rate, indicating excellent stability. Figure 5b shows the galvanostatic charge–discharge curves of rGO at different current densities (2–20 A g^−1^). All the charge–discharge curves possessed a symmetrical and linear triangle profile, implying that the rGO electrode had a rapid I–V response and charge–discharge reversibility. The specific capacitance of the rGO electrode at different charge–discharge current densities was calculated, as shown in Fig. 5c. We determined that the specific capacitance was about 110 F g^−1^ at a current density of 2 A g^−1^, along with 98 F g^−1^ at a current density of 20 A g^−1^. In addition, the stability of the capacitance of rGO was determined after 2000 cycles at a current density of 15 A g^−1^. As shown in Fig. 5d, the rGO electrode retained 98.5% of its initial specific capacitance after 2000 cycles and exhibited outstanding cycling stability.

**Fig. 6:**
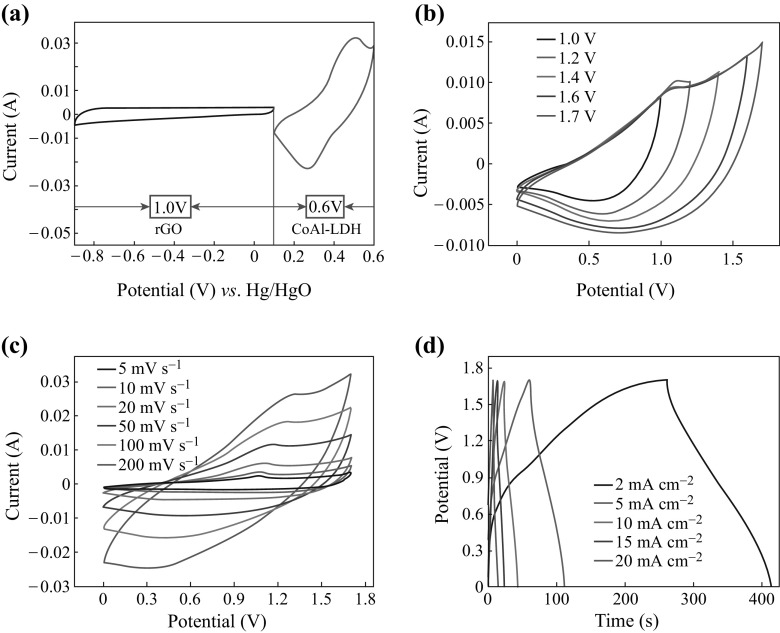
**a** Comparison of the CV curves of positive and negative electrodes; **b** CV curves of the ASC at different voltages; **c** CV curves of ASC device at different scan rates; **d** GCD curves of ASC device at different current densities

### Performance of ASC Based on CoAl-LDH and rGO Electrodes

A flexible ASC was fabricated by using CC@CoAl-LDH as the positive electrode, CC@rGO as the negative electrode, and KOH/PVA as the gelled electrolyte. To achieve the best electrochemical performance of ASC, the charge balance should be *q* ^+^ = *q* ^−^. *q* = *C* × Δ*E* × *m*, where *C* represents the specific capacitance of the electrode material, Δ*E* is the voltage window of the charge–discharge process, and m is the mass of active materials. To obtain *q* ^+^ = *q* ^−^, the mass of active materials on the electrode must be:

2 $$\frac{{m_{ + } }}{{m_{ - } }} = \frac{{C_{ - } \times \Delta E_{ - } }}{{C_{ + } \times \Delta E_{ + } }}$$

Hence, based on the value of the specific capacitance and voltage window for the CoAl-LDH and rGO electrodes, the optimal mass ratio between two electrodes was about 1:6.

**Fig. 7:**
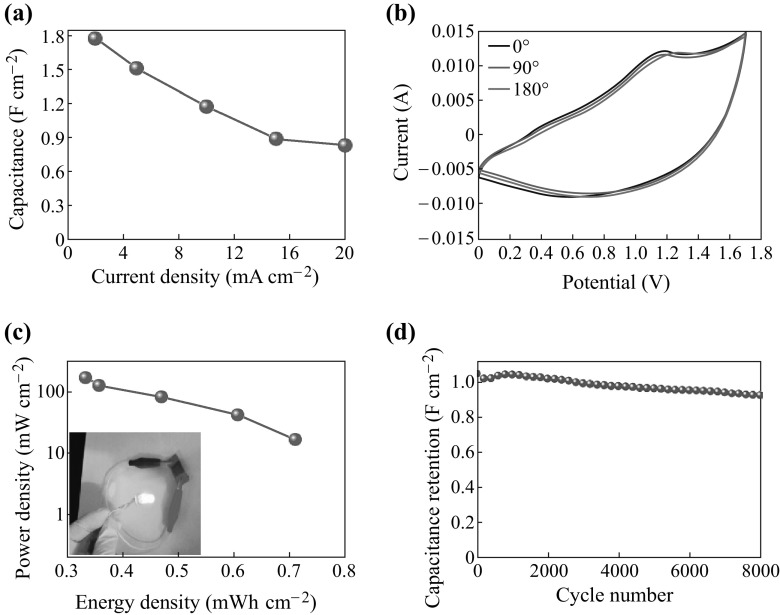
**a** The capacitance calculated from Fig. 6d; **b** CV curves (at 50 mV s^−1^) under different bending conditions; **c** Ragone plot of ASC device (*inset*: a *red* LED lighted by ASC device); **d** Cycle stability of the ASC device at a current density of 15 mA cm^−2^

Figure 6a shows a comparison of the CV curves of the positive (CoAl-LDHs) and negative (rGO) electrodes. It could be seen that the potential difference between the two electrodes was 1.6 V, and hence, the fabricated ASC could operate over a voltage window of 1.6 V. To verify this, the fabricated ASC was subjected to CV tests at different voltages from 1 to 1.7 V. As shown in Fig. 6b, the fabricated ASC revealed a stable capacitive behavior even when the voltage window reached up to 1.7 V. The enhanced performance could be ascribed to the synergistic effect between the CoAl-LDH and rGO electrodes. To further assess the performance of the ASC, CV and galvanostatic charge–discharge were performed. As shown in Fig. 6c, the flexible ASC exhibited rectangular CV curves, and the current clearly increased as the scan rate increases, implying excellent rate capability. All the GCD curves obtained with different current densities (2–20 mA cm^−1^) over a potential window of 1.7 V (Fig. 6d) showed typical triangular shapes, representing a well-balanced charge storage. The areal specific capacitance (Fig. 7a) of ASC calculated from the galvanostatic charge–discharge curves under different current densities was 1.77, 1.51, 1.17, 0.89, and 0.83 F cm^−2^ at 2, 5, 10, 15, and 20 mA cm^−2^.

The mechanical flexibility of the ASC is very important for wearable applications. Interestingly, no apparent changes in the CV curves were observed under different bending conditions (Fig. 7b). That is, the capacitance remained almost unchanged as the bending angles changed from 0° to 180°. These results proved the excellent mechanical flexibility of the ASC. Furthermore, the energy density and power density of the ASC are shown in a Ragone plot (Fig. 7c), which are calculated using the following equations:

3 $$E = 0.5 \times C_{\text{m}} \times (\Delta V)^{2}$$

4 $$P_{{\text{av}}} = \frac{E}{\Delta t}$$

where *C*_m_ (F cm^−2^) represents the specific capacitance of ASC, Δ*V* (V) is the operating voltage of the cell, Δ*t* (s) is the discharge time, *E* (Wh cm^−2^) is the energy density, and *P*_av_ (W cm^−2^) is the power density. The as-fabricated FASC devices showed a high energy density of 0.71 mWh cm^−2^ at a power density of 17.05 mW cm^−2^. As listed in Table 1, the CoAl-LDHs//rGO presented a high capacitance, energy density, and power density compared to others. In addition, the charged flexible ASC was able to power a commercial light-emitting diode (LED), as shown in the inset of Fig. 7c, implying the use of ASC for practical applications. The long-term cycling stability of the ASC device (Fig. 7d) was measured under galvanostatic charge–discharge at the current density of 15 mA cm^−2^. After 8000 cycles, the capacitance was reversibly maintained with only 7.1% loss of the initial value.

**Table 1:** The comparison of the capacitive performance of CoAl-LDHs//rGO ASC with others

ASC	Areal capacitance (F cm^−2^)	Voltage (V)	Energy density (mWh cm^−2^)	Power density (mW cm^−2^)	References
RGO@MnO_2_//RGO	0.34	1.5	0.0115	3.80	[38]
MnO_2_@PEDOT:PSS//AC	1.67	2.0	–	–	[39]
NiO//rGO	0.28	1.7	–	–	[40]
PPy@MnO_2_//AC	1.41	1.8	0.63	0.90	[41]
NiCoO_4_@Ni_3_S_2_//AC	2.25	1.8	–	–	[42]
CoAl-LDHs//rGO	1.77	1.7	0.71	17.05	This work

## Conclusion

A flexible asymmetric supercapacitor has been successfully fabricated by using CC@CoAl-LDHs as the positive electrode and CC@rGO as the negative electrode. PVA/KOH gel was used both as the electrolyte and the separator. Superior electrochemical properties of the flexible ASC were obtained, including high superficial specific capacitance of 1.77 F cm^−2^ at a current density of 2 mA cm^−2^, a wide operating potential of 1.7 V, and a high energy density of 0.71 mWh cm^−2^ at a power density of 17.05 mW cm^−2^. Importantly, this flexible ASC will find wide applications in portable electronic devices and hybrid vehicles.
